# Awareness and Acceptance of Pertussis Vaccination Among Pregnant Women in Taif Region, Saudi Arabia: A Cross-Sectional Study

**DOI:** 10.7759/cureus.41726

**Published:** 2023-07-11

**Authors:** Fatimah S Alshahrani, Ali N Elnawawy, Afnan M Alwadie

**Affiliations:** 1 Preventive Medicine, Ministry of Health, Taif, SAU; 2 Public Health and Community Medicine, Taif University, Taif, SAU; 3 Preventive Medicine, Saudi Board of Preventive Medicine, King Fahad Armed Forces Hospital, Khamis Mushait, SAU

**Keywords:** total awareness score, acceptance, awareness, newborns, pregnancy, pertussis vaccination, pertussis

## Abstract

Background and aim

Pertussis risk increases during pregnancy when it can be passed from an infected, non-vaccinated mother to the newborn. The pertussis vaccine, Boostrix (Tdap: tetanus, diphtheria, and pertussis), was approved by the U.S. Food and Drug Administration (FDA) for immunization of pregnant women during the third trimester. The current cross-sectional study aimed to assess the awareness level of pregnant women towards pertussis.

Methods

The study recruited 401 pregnant women from the Taif region through an interview questionnaire, which included different questions about the socio-demographic and clinical circumstances of the participants, as well as some variable parameters, to evaluate their awareness and acceptance of the pertussis vaccination.

Results

The statistical analysis of the data revealed that most participants were above 30 years of age (68.1%), urban (84.5%), highly educated (57.4%), and unemployed housewives (73.1%). Medical history showed that only 15 (3.7%) of participants had the vaccine during previous pregnancies. Analysis of the data showed a significant difference of limited awareness about the disease prognosis, infection, route of transmission, and vaccination (P<0.05). This was significant among the following participants: those in the age group >30 years, without a college degree, unemployed, in the third trimester of gestation, and with a medical history of pregnancy-related diseases. Furthermore, most participants at all educational levels didn’t significantly believe in the safety of the vaccine during pregnancy (P<0.05). Interestingly, most women who were without a college degree, unemployed, or not receiving a regular salary didn't intend to vaccinate their babies and neglected to obtain sufficient information about the vaccine or its side effects (P<0.05). The regression analysis revealed that women's age and education level were significant predictors of their total awareness score about the disease and its vaccination.

Conclusion

So, in conclusion, the study revealed that pregnant women's awareness of pertussis disease and its vaccine in the Taif region was associated with several demographic and clinical characteristics. Their acceptance of vaccination in relation to its safety or costs during pregnancy or to their infants depended significantly on their educational level and employment status. Therefore, the study recommends conducting more awareness campaigns about pertussis and its available vaccine. Also, more cross-sectional studies are required to measure the awareness of all women throughout the country.

## Introduction

Pertussis (whooping cough) is a dangerous infection that produces protracted bouts of coughing and choking, making breathing difficult. Pertussis is a highly contagious bacterial infection of the respiratory tract caused by Bordetella pertussis. The "whoop" is created by newborns gasping for air after each coughing attack, albeit this is not always the case [1]. It can lead to pneumonia or brain damage, particularly in babies. Most newborns with pertussis will require hospitalization, and in severe cases, they may die [2]. Pertussis rates have grown dramatically in recent years, and newborns who are too young to begin vaccines are especially vulnerable [2]. The annual report on the worldwide incidence of pertussis estimates nearly 48 million cases. Approximately 300,000 deaths due to pertussis infections have occurred in Africa in the last 10 years [3].

Pertussis is a vaccine-preventable disease. It can lead to high morbidity and mortality among unimmunized newborns. Routine pertussis immunization is advised during the first two months of life. After receiving the adult vaccine "Tdap" (tetanus, diphtheria, and acellular pertussis), pregnant women's immune systems produce protective antibodies, which they pass on to their offspring before delivery [4]. These antibodies provide neonates with short-term protection against pertussis infection. Immunization of pregnant mothers reduces the risk of illness in infants under two months old by 78%. Every pregnancy benefits from an early third-trimester immunization [5]. The optimal time to get vaccinated for infant protection is between 16 and 32 weeks of pregnancy, which plays a key role in protection against any further pertussis infection [2].

The Medicines and Healthcare Products Regulatory Agency (MHRA) found no indication of dangers to pregnancy or newborns in a trial of almost 20,000 vaccinated women [2]. Minor side effects may include local redness, edema, fever, loss of appetite, sleepiness, and agitation. The majority of these issues will resolve themselves. Other symptoms, such as high temperature, prolonged sobbing lasting more than three hours, fainting, and convulsions, are possible but less often. The vast majority of these reactions have no long-term repercussions [6]. The Centers for Disease Control and Prevention (CDC) recommend that pregnant women get vaccinated against pertussis as it might protect the newborns from severe infection in their early lives, which is unfortunately not taken into consideration in some obstetric clinics in Saudi Arabia [7].

In 2019, the Western region of Saudi Arabia accounted for 26.5% of the country’s total reported cases [8]. The Global Burden of Disease Studies showed that 24.1 million children aged less than five years were infected with pertussis worldwide in 2014, with over 160,000 pertussis deaths. The African region shared the most significant proportion (33% cases and 58% deaths). Of these, 21% of cases and 53% of deaths occurred in infants under 12 months of age [9]. Furthermore, in 2017, the World Health Organization (WHO) estimated 144,000 pertussis cases and 89,000 deaths among children less than five years old [10].

The Saudi Ministry of Health (MOH) pays full attention to maternal and child health, along with immunization programs. These efforts included providing vital educational information and health services in the maternity and childcare units of its health facilities. These educational services focus on the importance of reproductive health through attaining a safe pregnancy and childbirth period to ensure the health of both the mother and child. These services include ensuring an easy and free-of-complication pregnancy and children in good health. MOH has offered services such as calculating the best time to become pregnant, a predictable due date calculator, and a breastfeeding cycle [11].

Saudi MOH recommended hexavalent vaccines for babies at two, four, and six months of age, then two booster doses at 18 months of age, and at preschool entry [12]. Moreover, MOH has developed the Mother and Child Health Passport System. This system keeps records of illness history, follows up on the health status of mother and child, and requires examinations and analyses [11]. Moreover, it is recommended that pregnant women take the adult vaccine combination Tdap in the third trimester of pregnancy (27-36 weeks) in every pregnancy [13].

 For all the reasons mentioned above, it is important to survey the awareness and factors affecting acceptance of pertussis vaccination during pregnancy to address the problem and resolve these issues to protect new babies from infection and its complications. So, the current study aimed to assess the knowledge and acceptance of pregnant women in the Taif region, Saudi Arabia, towards the pertussis vaccine during pregnancy.

## Materials and methods

Study design and population

The current cross-sectional study included 401 randomly selected pregnant women attending obstetric clinics at the King Faisal Medical Complex in Taif, Saudi Arabia. King Faisal Medical Complex is the only hospital related to the Saudi MOH with obstetric clinics performing the main three ultrasounds required for pregnancy follow-up. The study period was from March 2023 to June 2023 and was conducted using an interview questionnaire.

Inclusion criteria

The inclusion criteria for recruitment included pregnant women aged more than 18 years, who attended a follow-up consultation and agreed to participate in the study after completing an informed consent.

Exclusion criteria

The exclusion criteria comprised pregnant women who refused to complete the survey and provide consent to participate. The high or low obstetric scores weren't included in participants' selection as a measurable factor of awareness or acceptance.

Ethical considerations

The study received institutional ethical approval from the Institutional Review Board at King Faisal Medical Complex in Taif for Science and Technology (IRB registration number: H-02-T-123 and approval number: 2023-B-18).

Participants selection and sample size calculation

We used the systematic sampling method. We selected pregnant women from a list of women who attended checkups in this facility and chose the first woman randomly from the first five women; then we chose one after every two women from this list. The sample size was calculated according to the following equation: n= (z^2^) P(100-P)/d^2^ where n = sample size, z = z statistic for the level of confidence (for the conventional 95% confidence interval, z value is 1.96), P = 0.5, and d = margin of error (set as 5%). The minimum sample size required was 385. We selected 401 pregnant women to participate in this study. 

We used an interview questionnaire that was adapted from previous studies [8, 14]. The necessary modifications were made to make it suitable for our study. Modifications were made based on the experience of the researcher and the consultation of experts working in this field. The questionnaire was divided into two sections (discussed below). We conducted a pilot trial on 25 subjects (removed from the final analysis) to test the objectivity of the questionnaire or if any amendments were required.

 Structure of the questionnaire

Section I

This section included the demographic characteristics and the clinical history of participants comprising 11 variables: age, residency, marital status, education level, employment status, regular payment or salary, gestational stage, previous pregnancy-induced diseases, preexisting diseases, total pregnancies, and total births.

Section II

This section included 15 closed-ended questions with yes or no options that tested the awareness of participants about their general knowledge about the pertussis disease, the vaccine, its effect on pregnancy, vaccine cost and availability, vaccine safety, and vaccine side effects. 

Reporting of data and statistical analysis

After completion of data collection, the data were coded, cleaned, and entered into an Excel sheet and then exported to SPSS Statistics software v.26 (IBM, Armonk, NY). Mean and standard deviation were used to describe the quantitative variables while numbers and percentages were used to describe the qualitative variable. The chi-square (χ2) test was used for associations between categorical variables and correlations were calculated by computing Cramér's V (φc) between the nominal variables at the degree of freedom (df). Cramér's V (φc) measures the effect size (ES) for the chi-square test of independence, where ES ≤ 0.21 describes weak association, 0.21 < ES ≤ 0.35 describes moderate association, and ES > 0.35 describes strong association at df=2 [15]. Multiple regression analysis was used to assess the impact of the independent variables after adjustment on the total awareness score at 95% confidence intervals (CI). At a p-value less than 0.05, differences were judged statistically significant.

## Results

In the current study, 401 pregnant women consented to participate in the study and answered the questionnaire. As shown in Table 1, most of them were above 30 years old (68.1%), living in cities of the Taif region (84.5%), and were highly educated (57.4%). On the other hand, most of them were unemployed housewives (73.1%) and didn’t receive regular salaries or payments (65.6%). Also, the majority of participants were in their third trimester of pregnancy (57.1%) and their clinical history didn’t refer to any previous (91.8%) or current (84.3%) pregnancy-related disorders (such as hypertension, diabetes, cardiac diseases, etc).

**Table 1:** Socio-demographic and clinical characteristics of the study population (N=401).

Variables		Frequency N (%)
Age group (year)	18-20	11 (2.7%)
	21-29	117 (29.2%)
	≥ 30	273 (68.1%)
Type of residence	City	339 (84.5%)
	Village	62 (15.5%)
Marital status	Married	389 (97%)
	Divorced	8 (2%)
	Widowed	4 (1%)
Educational level	Primary school	38 (9.5%)
	Middle / Secondary school	133 (33.2%)
	Bachelor's degree or higher	230 (57.4%)
Employment status	Employee	90 (22.4%)
	Executive	4 (1%)
	Health-care professionals	14 (3.5%)
	Unemployed	293 (73.1%)
Regular payment/salary	Yes	138 (34.4%)
	No	263 (65.6%)
Gestational stage	First trimester	63 (15.7%)
	Second trimester	109 (27.2%)
	Third trimester	229 (57.1%)
Pregnancy-induced disease	Yes	63 (15.7%)
	No	338 (84.3%)
Preexisting diseases	Yes	33 (8.2%)
	No	368 (91.8%)
Total pregnancies (Median (Min.-Max.))		3 (1-13)
Total births (Median (Min.-Max.))		2 (0-9)

The analysis of data obtained from Section II in the study questionnaire is included in Table 2. It was reported that most of the participants ignored basic information about the nature of pertussis disease or its vaccine efficacy. We noted that 28 (7%) of the participants were recommended to have the pertussis vaccine, mainly doctors and healthcare professionals (HCP), whereas seven (1.7%) were discouraged, mainly by relatives and friends, from taking it. Furthermore, most of them doubted the safety of the pertussis vaccination during pregnancy; only 15 (3.7%) women took the pertussis vaccine during previous pregnancies. Also, we noticed that the availability of the vaccine at no cost didn’t change the opinion of 55.1% of participants who refused to be vaccinated during pregnancy. Similarly, 48.9% of participants refused to vaccinate their babies with the vaccine free of charge, compared to 56.9% who refused to vaccinate them at their own expense. Finally, most of them didn’t know about the expected side effects or safety levels of the pertussis vaccine during pregnancy or to their children. 

**Table 2:** Investigation inquires of awareness points about pertussis disease and vaccination among the study participants (N=401).

Q	Category	Awareness point		N (%)
1	Pertussis disease	Have you ever heard about pertussis?	Yes	169 (42.1%)
			No	232 (57.9%)
2		Do you know that pertussis is an infectious disease?	Yes	118 (29.4%)
			No	283 (70.6%)
3		Do you know the route of transmission? (Respiratory droplet)	Yes	91 (22.7%)
			No	310 (77.3%)
4		Did you know any baby becomes infected with pertussis in the first two months after delivery?	Yes	11 (2.7%)
			No	390 (97.3%)
5	Pertussis vaccine	Do you know there is a vaccine for protection from pertussis?	Yes	101 (25.2%)
			No	300 (74.8%)
6		Has anyone recommended you take pertussis vaccine?	Yes	28 (7%)
			No	373 (93%)
7		Has anyone discouraged you from taking pertussis vaccine?	Yes	7 (1.7%)
			No	394 (98.3%)
8		Do you know about safety and side effects of pertussis vaccine?	Yes	43 (10.7%)
			No	358 (89.3%)
9	Pertussis vaccine and pregnancy	Did you take pertussis vaccine during previous pregnancy if present?	Yes	15 (3.7%)
			No	386 (96.3%)
10		Do your belief pertussis vaccine more dangerous in pregnant women than nonpregnant women?	Yes	123 (30.7%)
			No	278 (69.3%)
11		Do you know that taking the pertussis vaccine will protect newborn from getting pertussis?	Yes	75 (18.7%)
			No	326 (81.3%)
12	Pertussis vaccine cost/availability	If a vaccine was available for free, would agree to receive the vaccine during pregnancy?	Yes	180 (44.9%)
			No	221 (55.1%)
13		Do you agree to take pertussis vaccine during pregnancy at your expense?	Yes	135 (33.7%)
			No	266 (66.3%)
14		After the baby is born, will you vaccinate the baby at your own expense?	Yes	173 (43.1%)
			No	228 (56.9%)
15		After the baby is born, will you allow the baby to be vaccinated for free?	Yes	205 (51.1%)
			No	196 (48.9%)

Analysis of the relations between different demographic and clinical characteristics and general knowledge about pertussis and its vaccine are shown in Table 3. For participant ages, the analysis revealed a significant medium association with their general knowledge about the disease (P<0.001, df=2, φc=0.244), and a weak association with their general knowledge about its infection (P=0.002, df=2, φc=0.176) and route of transmission (P<0.001, df=2, φc=0.186). Regarding education level, the statistical analysis showed a significant medium association with their general knowledge about the disease (P<0.001, df=2, φc=0.264), its infection (P<0.001, df=2, φc=0.255), and a weak association with their general knowledge about and route of transmission (P=0.007, df=2, φc=0.156). For employment status, the statistical analysis showed a significant weak association with their general knowledge about the disease (P=0.004, df=2, φc=0.182), medium association with their general knowledge about its infection (P<0.001, df=2, φc=0.218), route of transmission (P<0.001, df=2, φc=0.288), its vaccine (P<0.001, df=2, φc=0.244), and the vaccine side effects (P<0.001, df=2, φc=0.423).

**Table 3:** Association between the awareness about pertussis diseases and vaccination and different socio-demographic and clinical characteristics of the study participants (N=401). HCP: Health care professional; #Chi-square (χ2); *Significant; ^ Cramer's V value.

Variables		Pertussis disease (basic knowledge)				Pertussis Vaccine (basics knowledge)			
		General	Infectious	Trans. route	Newborn infection	General	Advice to	Advice not to	Side effects
Age (years)	18-20	0 (0%)	0 (0%)	0 (0%)	1 (0.2%)	1 (0.2%)	0 (0%)	1 (0.2%)	0 (0%)
	21-29	33 (8.2%)	24 (6%)	15 (3.7%)	3 (0.7%)	24 (6%)	8 (2%)	2 (0.5%)	8 (2%)
	≥ 30	136 (33.9%)	94 (23.4%)	76 (19%)	7 (1.7%)	76 (19%)	20 (5%)	4 (1%)	35 (8.7%)
	P-value#	<0.001*	0.002*	<0.001*	0.426	0.143	0.644	0.166	0.11
	Correlation^	0.244	0.176	0.186	0.065	0.098	0.047	0.095	0.105
Education level	Primary	7 (1.7%)	6 (1.5%)	7 (1.7%)	1 (0.2%)	5 (1.2%)	2 (0.5%)	1 (0.2%)	2 (0.5%)
	Secondary	40 (10%)	24 (6%)	19 (4.7%)	2 (0.5%)	29 (7.2%)	5 (1.2%)	1 (0.2%)	7 (1.7%)
	Higher	122 (30.4%)	88 (21.9%)	65 (16.3%)	8 (2%)	67 (16.8%)	21 (5.3%)	5 (1.2%)	34 (8.5%)
	P-value#	<0.001*	<0.001*	0.007*	0.54	0.06	0.14	0.553	0.056
	Correlation^	0.264	0.255	0.156	0.055	0.118	0.099	0.054	0.01
Employment status	Employee	44 (11%)	34 (8.5%)	28 (7%)	2 (0.5%)	28 (7%)	8 (2%)	1 (0.2%)	11 (2.7%)
	Executive	3 (0.7%)	2 (0.5%)	1 (0.2%)	0 (0%)	1 (0.2%)	0 (0%)	0 (0%)	0 (0%)
	HCP	11 (2.7%)	10 (2.5%)	11 (2.7%)	0 (0%)	10 (2.5%)	0 (0%)	0 (0%)	11 (2.7%)
	None	111 (27.7%)	72 (17.9%)	5 (12.7%)	9 (2.2%)	62 (15.5%)	20 (5%)	6 (1.5%)	21 (5.3%)
	P-value#	0.004*	<0.001*	<0.001*	0.869	<0.001*	0.601	0.876	<0.001*
	Correlation^	0.182	0.218	0.288	0.042	0.244	0.068	0.041	0.423
Gestational trimester	First	18 (4.5%)	15 (3.7%)	13 (3.2%)	0 (0%)	17 (4.2%)	3 (0.7%)	1 (0.2%)	8 (2%)
	Second	34 (8.5%)	22 (5.5%)	17 (4.2%)	2 (0.5%)	23 (5.7%)	6 (1.5%)	3 (0.7%)	7 (1.7%)
	Third	117 (29.2%)	81 (20.2%)	61 (15.2%)	9 (2.2%)	61 (15.2%)	19 (4.7%)	3 (0.7%)	28 (7%)
	P-value#	<0.001*	0.009*	0.07	0.19	0.514	0.483	0.636	0.234
	Correlation^	0.21	0.153	0.115	0.091	0.058	0.06	0.048	0.085
pregnancy-induced diseases	Yes	36 (9%)	27 (6.7%)	23 (5.7%)	1 (0.2%)	19 (4.7%)	5 (1.2%)	3 (0.7%)	11 (2.7%)
	P-value#	0.009*	0.011*	0.004*	0.541	0.322	0.746	0.046*	0.06
	Correlation^	0.131	0.127	0.142	0.031	0.049	0.016	0.099	0.094
Preexisting diseases	Yes	18 (4.5%)	13 (3.2%)	13 (3.2%)	2 (0.5%)	9 (2.2%)	4 (1%)	2 (0.5%)	4 (1%)
	P-value#	0.132	0.19	0.017*	0.223	0.773	0.227	0.048*	0.786
	Correlation^	0.075	0.065	0.119	0.061	0.014	0.06	0.099	0.014

For clinical characteristics (Table 3), the statistical analysis revealed a medium association between gestational trimester and basic knowledge about disease (P=<0.001, df=3, φc=0.21) and a weak association with basic knowledge about its infection (P=0.009, df=3, φc=0.153). Regarding the co-morbidities induced during pregnancy, there were weak associations with participants’ general knowledge about the disease (P=0.009, df=1, φc=0.131), its infection (P=0.011, df=1, φc=0.127), route of transmission (P=0.004, df=1, φc=0.142), and discouragement against vaccination (P=0.046, df=1, φc=0.099). Furthermore, for preexisting diseases, there was a weak association between participants’ awareness about the route of transmission (P=0.017, df=1, φc=0.119) and discouragement against vaccination (P=0.048, df=1, φc=0.099). We didn’t find any significant association between these variables and the participants’ awareness about the possibility of newborn infection with pertussis, two months post-delivery or the effect of others’ advice to have the vaccine.

Another comparison was performed between the demographic and clinical characteristics of the study participants and their thoughts about the effect of the vaccine on pregnancy and newborns. Also, we studied the effect of vaccine cost on their acceptance to be vaccinated during pregnancy or vaccination of their newborns (Table 4). The results didn’t reveal any association with age, gestational trimester, or the co-morbidities induced during the pregnancies of participants. Also, no association was found between the records of previous pregnancy vaccination and any of the variables. On the other hand, weak associations were found between the education level and participants’ worries about the safety of vaccine during pregnancy (P=0.03, df=2, φc=0.132), their acceptance of free or paid vaccine during pregnancy (P=0.001 and 0.015, df=2, φc=0.132 and 0.145, respectively), or to their new babies (P<0.001, df=2, φc=0.2 and 0.207, respectively).

**Table 4:** Association between the demographic and clinical characteristics of the study participants and the safety and cost of the vaccination of pregnant mothers and newborns (N=401). HCP: Health care professional; #Chi-square (χ2); *Significant; ^ Cramer's V value.

Variables		Pertussis vaccine and pregnancy			Effect of pertussis vaccine cost on the acceptance to give it to			
					Pregnant woman		Newborn	
		Previous vaccination	Safety to pregnancy	Newborn protection	Free	Paid	Paid	Free
Age (years)	18-20	1 (0.2%)	4 (1%)	0 (0%)	3 (0.7%)	1 (0.2%)	3 (0.7%)	4 (1%)
	21-29	4 (1%)	34 (8.5%)	19 (4.7%)	57 (14.2%)	41 (10.2%)	51 (12.7%)	57 (14.2%)
	≥ 30	10 (2.5%)	85 (21.2%)	56 (14%)	120 (29.9%)	93 (23.2%)	119 (29.7%)	144 (35.9%)
	P-value#	0.634	0.844	0.166	0.338	0.213	0.56	0.468
	Correlation^	0.048	0.029	0.095	0.047	0.088	0.054	0.062
Education level	Primary	1 (0.2%)	6 (1.5%)	6 (1.5%)	11 (2.7%)	10 (2.5%)	9 (2.2%)	9 (2.2%)
	Secondary	1 (0.2%)	36 (9%)	18 (4.5%)	48 (12%)	34 (8.5%)	46 (11.5%)	62 (15.5%)
	Higher	13 (3.3%)	81 (20.2%)	51 (12.7%)	121 (30.2%)	91 (22.7%)	118 (29.4%)	134 (33.4%)
	P-value#	0.056	0.03*	0.112	0.001*	0.015*	<0.001*	<0.001*
	Correlation^	0.12	0.132	0.104	0.184	0.145	0.2	0.207
Employment status	Employee	3 (0.7%)	31 (7.7%)	21 (5.2%)	46 (11.5%)	33 (8.2%)	45 (11.2%)	48 (12%)
	Executive	0 (0%)	1 (0.2%)	1 (0.2%)	2 (0.5%)	2 (0.5%)	3 (0.7%)	3 (0.7%)
	HCP	1 (0.2%)	6 (1.5%)	10 (2.5%)	10 (2.5%)	10 (2.5%)	11 (2.7%)	12 (3%)
	None	11 (2.7%)	85 (21.3%)	43 (10.8%)	122 (30.4%)	90 (22.5%)	114 (28.5%)	142 (35.4%)
	P-value#	0.886	0.568	<0.001*	0.083	0.012*	0.005*	0.035*
	Correlation^	0.04	0.071	0.274	0.129	0.165	0.178	0.147
Gestational trimester	First	1 (0.2%)	18 (4.5%)	14 (3.5%)	28 (7%)	19 (4.7%)	27 (6.7%)	32 (8%)
	Second	3 (0.7%)	27 (6.7%)	15 (3.7%)	49 (12.2%)	35 (8.7%)	42 (10.5%)	48 (12%)
	Third	11 (2.8%)	78 (19.5%)	46 (11.5%)	103 (25.7%)	81 (20.2%)	104 (25.9%)	125 (31.2%)
	P-value#	0.401	0.207	0.279	0.997	0.683	0.49	0.193
	Correlation^	0.067	0.089	0.08	0.004	0.044	0.06	0.091
Pregnancy-induced diseases	Yes	3 (0.7%)	23 (5.7%)	12 (3%)	31 (7.7%)	22 (5.5%)	31 (7.7%)	34 (8.5%)
	P-value#	0.642	0.274	0.939	0.453	0.818	0.29	0.623
	Correlation^	0.023	0.055	0.004	0.037	0.011	0.053	0.025
Preexisting diseases	Yes	1 (0.2%)	17 (4.2%)	9 (2.2%)	16 (4%)	13 (3.2%)	12 (3%)	18 (4.5%)
	P-value#	0.822	0.007*	0.188	0.665	0.467	0.412	0.681
	Correlation^	0.011	0.135	0.066	0.022	0.036	0.041	0.021

Finally, the regression analysis revealed that two independent variables might be considered as predictors for the total awareness score about pertussis disease and acceptance of its vaccination during pregnancy. Here, the variables were age (P=0.026; 95% CI: 0.088-1.363) and educational level (P<0.001; 95% CI: 0.574-1.652) (Table 5).

**Table 5:** The multiple regression analysis of the significant prediction of the impact of different demographics or clinical characteristics on the total awareness score of pertussis and its vaccination. t: t-test; *Significant; R: multiple regression; SE: standard error of estimates

Variable	Standardized Coefficient B	t	Sig.	95.0% CI B		R	R^2^	SE
				Lower Bound	Upper Bound			
Age	0.113	2.237	0.026*	0.088	1.363	0.329	0.108	3.25408
Education level	0.217	4.058	<0.0001*	0.574	1.652			
Employment status	0.021	0.339	0.743	-0.278	0.394			
Gestational stage	0.061	1.229	0.220	-0.166	0.720			
Pregnancy-induced diseases	0.056	1.13	0.259	-0.389	1.439			
Pre-existing diseases	0.033	0.665	0.507	-0.799	1.616			

**Figure 1:**
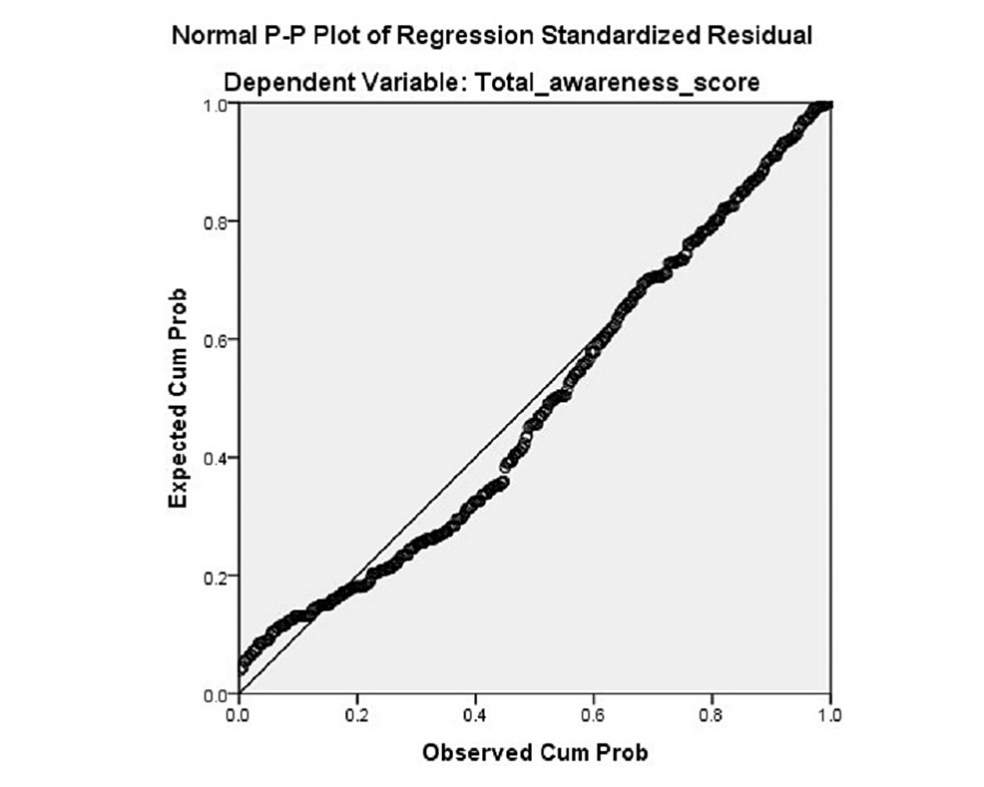
Normal P-P plots of regression standardized residuals to assess the impact of the independent variables after adjustment on total awareness score.

To summarize the regression analysis results, the normal P-P plots of regression standardized residuals are shown in Figure 1. It indicated strong regression, where the data are expressed closer to the diagonal line. That indicated the residuals are normally distributed and the regression is successful. 

## Discussion

According to the annual report on the global incidence of pertussis, over 48 million new infections were projected to occur in Africa over the last decade [16]. Also, WHO reports from over the past 10 years revealed high prevalence and mortality rates among children under the age of five years due to pertussis infection [10].

In the current study, the sociodemographic and clinical characteristics of 401 pregnant Saudi women from the Taif region were studied for their awareness of pertussis and its vaccine. The analysis and interpretations of the results highlighted the impact of age, education, occupation, and monthly income on the participants' awareness of the disease and vaccine. In agreement with these findings, a cross-sectional study in Palermo, Italy, reported a significant correlation between employed pregnant women with higher educational levels and their awareness and willingness to be vaccinated against pertussis [17]. Another cross-sectional study from Victoria, Australia, revealed that most mothers (75%) with ages higher than 33, with higher educational levels (46%), and in households with higher incomes (more than $95,000) were aware of pertussis, and 70% accepted vaccination during pregnancy or after delivery [18]. Also, the study reported that the number of urban parents who accepted vaccination was lower than that of rural parents [18]. A Canadian study questioned 5091 mothers who gave birth between September 2018 and March 2019 regarding pertussis immunization during their pregnancy. Independent characteristics related to non-vaccination, according to this study, included being born outside of Canada, having a low income, living in a province where pertussis vaccination was not free, having previous live births, and obtaining maternity care from a midwife [19]. This is likely in accordance with our findings.

In the current study, 96.3% of the studied cases reported that they didn’t receive any pertussis vaccine during previous pregnancies, and 48.9% of them refused to vaccinate their newborns, even with a cost-free vaccine. An interesting study from Riyadh, Saudi Arabia of 258 pregnant women (28-45 years old) revealed that 90.69% of them didn’t receive any doses of the pertussis vaccine and were at risk of infection [20]. Another study from Buenos Aires, Argentina, reported that 92% of infants born to vaccinated mothers were immune against pertussis infection [21].

In the current study, the multiple linear regression analysis revealed a strong impact of pregnant women’s age and education levels as predictors of future awareness of the disease or acceptance of the vaccination. In contrast to our findings, a Spanish cross-sectional study reported that pre-existing hypertension or HIV infection was negatively associated with Tdap vaccination [22]. Another study from Bangkok, Thailand, reported that the decisions of doctors and husbands had the highest impact on the intention to receive pertussis vaccination during pregnancy and were thought to be safe [23]. In China, factors influencing low pertussis vaccine awareness among pregnant women included employment as nonmedical-institution employees, a lower educational level, pregnancy stage beyond the first trimester, and not carrying children. Pertussis exhibited a lower degree of awareness among pregnant women as compared to influenza illness. As a result, it may also be a source of low pertussis vaccination knowledge [14]. In the United Arab Emirates, substantial sero-negativity for pertussis vaccine among pregnant women necessitates the inclusion of pertussis vaccination in the national immunization program [24]. So, predictors of acceptance or awareness about the importance of pertussis vaccination might differ from one society to another, which might be due to different reasons such as education types, percentage of unemployment, average individual income, the prevalence of common diseases and pandemics.

In Japan, the maternal (Tdap) vaccine is not approved by the government, and the Japanese national immunization program also does not include it. Most Japanese pregnant women wanted the government to approve maternal Tdap immunization [25]. In Ireland, there was low awareness about pertussis and influenza vaccines during pregnancy. And this was attributed to inadequate knowledge of immunization guidelines among healthcare professionals, a lack of awareness of the need to get the vaccines, and poor communication [1]. In China, the factors affecting low knowledge about the pertussis vaccine among pregnant women were occupations as nonmedical-institution staff, lower educational level, pregnancy stage past the first trimester, and not bearing children. In comparison to awareness about influenza infection, pertussis had a lower level of awareness among pregnant women [14].

In the Arabian Peninsula, a cross-sectional study was conducted in Riyadh to explore the seroprevalence of pertussis among pregnant women, it was revealed that most of the tested women were seronegative and non-immunized against pertussis infection despite the Saudi program of immunization [20]. Furthermore, there was poor knowledge about vaccination during pregnancy in a study conducted in the Al Ahsaa region [26]. In the United Arab Emirates, high seronegative for the pertussis vaccine among pregnant women makes it necessary to introduce the pertussis vaccination of pregnant women into the national immunization program [24].

In general, safety concerns, a lack of information among pregnant women, and a lack of confidence among healthcare practitioners to discuss immunization are significant barriers to vaccination adoption.

## Conclusions

In conclusion, the current study showed that the awareness of pregnant women in Taif region, Saudi Arabia, about pertussis risk and the importance of its available vaccine was affected by their education level, age, and employment status. Furthermore, the ages and educational levels of pregnant women were significant predictors to assess the impact of the independent variables after adjustment on the total awareness score.

The relatively small sample size and the enrollment of women in a single hospital were some of the limitations that should be considered, as the results may differ according to the area because of differences in the prevalence of infection and community education levels. So, results may not be generalized. 

For all of the reasons stated above, it is recommended to study other variables influencing the acceptability of pertussis vaccination during pregnancy to address and resolve the issue and prevent newborns from illness and its implications. Also, more additional pertussis awareness efforts among pregnant women should be done in hospitals, schools, educational institutions, and public areas. Future large cohort studies should be conducted to accurately reflect the total awareness and acceptance of all pregnant women in Saudi Arabia, dependent on the above-mentioned factors.
